# The history of Chagas disease

**DOI:** 10.1186/1756-3305-7-317

**Published:** 2014-07-10

**Authors:** Dietmar Steverding

**Affiliations:** 1 BioMedical Research Centre, Norwich Medical School, Norwich Research Park, University of East Anglia, Norwich NR4 7TJ, UK

**Keywords:** Chagas disease, American trypanosomiasis, History, *Trypanosoma cruzi*

## Abstract

The ancestor of *Trypanosome cruzi* was probably introduced to South American via bats approximately 7-10 million years ago. When the first humans arrived in the New World, a sylvatic cycle of Chagas disease was then already well established. Paleoparasitological data suggests that human American trypanosomiasis originated in the Andean area when people founded the first settlements in the coastal region of the Atacama Desert. Identification of *T. cruzi* as the etiological agent and triatome bugs as the transmission vector of Chagas disease occurred within a few years at the beginning of the 20^th^ century. History also teaches us that human activity leading to environmental changes, in particular deforestation, is the main cause for the spread of Chagas disease. Recently, migration of *T. cruzi*-infected patients has led to a distribution of Chagas disease from Latin America to non-endemic countries in Europe, North America and western Pacific region.

## Background

Chagas disease or American trypanosomiasis is a zoonotic infectious disease affecting humans in Latin America. The disease is caused by the protozoan flagellate *Trypanosoma cruzi* that lives and multiplies within cells from a variety of tissues. The parasite is usually transmitted via the faeces of blood-sucking bugs belonging to the subfamily Triatominae (kissing bugs or conenose bugs), with *Triatoma infestans*, *Rhodnius proxilus* and *Panstrongylus megistus* being the most important vectors. As *T. cruzi* cannot penetrate intact skin, it enters the human body through microlesions that have been introduced and contaminated with faeces when individuals scratch the itching vector’s bite. In addition, *T. cruzi* is able to break through intact mucous membranes (such as the conjunctiva and the gastric epithelium). Other modes of transmission include blood transfusion, organ transplantation, via breast milk, congenitally via the placenta and by ingestion of contaminated food and drink. Chagas disease is endemic in all South and Central American countries as well as in Mexico [1]. In addition, the southern half of the United States contains enzootic cycles of *T. cruzi* and autochthonous vector-borne human infections have been reported in Texas, California, Tennessee, Louisiana and Mississippi [2,3].

There are two phases during the course of Chagas disease. The acute phase begins 6-10 days after infection and lasts for about 4-8 weeks [4]. In most cases, the acute phase passes unnoticed as the clinical symptoms are nonspecific (including fever, hepatosplenomegaly or lymphadenopathy) and are typical for many infections [5]. As a specific symptom, inflammatory oedema at the site of infection may be observed. If the entry site is the skin, the swelling is known as a chagoma. In case the entry point is the eye, a unilateral periorbital swelling, a so-called Romaña’s sign, may develop. Acute myocarditis and acute meningoencephalitis can be occasionally seen in young children aged 1-5 years and is in most cases fatal [4]. Abnormalities in the ECG are observed in about 50% of cases which usually disappear later in the disease course [6]. During the acute phase, circulating trypanosomes can be easily found in the blood of patients [5]. Eventually, the parasite and the host reach an immunological balance and the disease enters the chronic phase. In this phase, the parasitaemia is greatly reduced and the patients become asymptomatic. Most patients remain in a so-called indeterminate (latent) chronic stage for the rest of their lives and do not develop any chronic symptoms. However, 15-30% of infected people will enter the determinate (symptomatic) chronic stage of the disease associated with the manifestation of organ damage [5]. This usually happens 10-25 years after the initial infection [5]. Typical manifestations are the dilatation of the heart (chronic chagasic cardiomyopthy) and/or parts of the digestive track (megaoesophagus and megacolon). Although the clinical manifestation of megaoesophagus and megacolon can be extremely debilitating conditions for many patients, the development of cardiomyopathy is a life-threatening problem in most cases. The different pathologies of Chagas disease are caused by six discrete typing units (DTUs) of *T. cruzi* (TcI-TcVI), which have distinct geographical distributions and extensive genetic diversity [7].

In contrast to African trypanosomes which were important selective factors in the human evolution [8], American trypanosomes affected human beings only recently who arrived in the New World less than 15,000 years ago [9].

## Review

### Origin of *T. cruzi*

Phylogenetic analysis of 18S rRNA sequences indicates that salivarian trypanosomes (the *T. brucei* clade grouping those trypanosomes that are transmitted by bites) diverged from the stercorarian trypanosomes (*T. cruzi* clade grouping those trypanosomes that are transmitted by faecal contamination) approximately 100 million years ago [10]. As at the same time South America, Antarctica and Australia separated from Africa, it was suggested that *T. cruzi* and related trypanosomes evolved in isolation in early terrestrial mammals [11]. This idea is known as the southern super-continent hypothesis. Based on this scenario one would expect a high diversity of *T. cruzi* clade trypanosomes in South American terrestrial mammals provided that they had been present on the continent since the break up the southern super-continent 40 million years ago [11]. However, this is not the case. No *bona fide* species have been discovered in the *T. cruzi* clade from any South American terrestrial mammal to date [11], i.e., no co-evolution generating host species specific genotypes has occurred. In addition, as *T. cruzi* clade trypanosomes are also present in land mammals from Africa and Australia [11], the role of geographical isolation in the evolution of *T. cruzi* is questionable.

Recent molecular evidence indicates that *T. cruzi* has evolved from a bat trypanosome, a scenario known as the bat seeding hypothesis [11]. This idea is supported by the fact that the closest genetically characterised relative of *T. cruzi* is *T. marinkellei* from South American bats [10,12-14]. Both diverged approximately 6.5-8.5 million year ago [15,16] and could be regarded as subspecies (i.e. *T. c. cruzi* and *T. c. marinkellei*) [17]. The recently described *T. erneyi* and *T. livingstonei* found in bats from Mozambique [18,19], and *T. dionisii* from Old and New World bats [10,12,14,20] are also close relatives of *T. cruzi*. Moreover, *T. cruzi* has been detected in South American bats [12,21,22] with one specific genotype, TcBat, only found in bats so far [23]. TcBat is most closely related to *T. cruzi* TcI which primarily is associated with opossums and conenose bugs of the genus *Rhodnius* in arboreal ecotopes [11]. Based on these facts it is reasonable to suppose that the common ancestor of the members of the *T. cruzi* clade was a bat trypanosome. Presumably, trypanosome-infected bats have colonised South America about 7-10 million years ago via North America [24]. Then, various independent bat trypanosome lineages switched from bats into terrestrial mammals probably facilitated by invertebrate vectors feeding on both bats and terrestrial mammals living in the same arboreal ecotopes [10]. One such switch gave rise to *T. cruzi* in the Pliocene [25]. The diversification of *T. cruzi* into the current DTU lineages TcI-TcVI and TcBat started quite recent about 1-3 million years ago [25].

### Pre-Columbian time

There is evidence that soon after having populated South America humans became infected with *T. cruzi*. The earliest detection of a *T. cruzi* infection in a human comes from a 9000 year old Chinchorro mummy through PCR amplification of kinetoplasid DNA sequences [26]. The Chinchorros were the first people identified to settle along South American’s coastal region of the Atacama Desert in southern Peru and northern Chile. *T. cruzi* infections were also found in mummies of subsequent cultures that succeeded the Chinchorros and were living in the same area up to the time of the Spanish conquest in the 16^th^ century [26]. The prevalence rate for *T. cruzi* infection in these populations was 41% without any significant differences among the individual cultures indicating that already in pre-Columbian time Chagas disease was widely spread in civilised societies [26]. Infections with *T. cruzi* were also detected in human remains from other archaeological excavation sites in America [27]. For example, *T. cruzi* DNA was found in a 560 year old partially mummified human body and in a 4,500-7,000 year old human bone fragment both unearthed in the Peruaçu Valley in the State of Minas Gerais, Brazil [28,29]. Another case of a prehistoric *T. cruzi* infection was reported in a 1,150 year old mummy recovered from the Chihuahuan Desert near the Rio Grande in Texas [27]. In addition to the detection of *T. cruzi* in human remains, several exhumed mummies also showed clinical signs of Chagas disease [26-28,30]. Further evidence of American trypanosomiasis in Pre-Columbian times comes from Peruvian ceramics dated to the 13^th^-16^th^ centuries showing possible representations of Chagas disease [31]. This also included a head with a unilateral swelling of the eyelid reminiscent of the Romaña’s sign [31].

Based on the paleoparasitological data, it has been hypothesised that Chagas disease originated in the Andean region [32]. It is believed that the Chinchorro people were the first to leave a nomadic lifestyle and to settle down to start arable farming and livestock breeding [26,30,31]. Upon settlement, prehistoric people intruded and participate in the sylvatic cycle of *T. cruzi*, and gradually a domestic cycle of transmission of Chagas disease emerged [26,31,32]. The development of a domestic *T. cruzi* transmission cycle was facilitated by the ability of some species of triatomine bugs, in particular *T. infestans*, to adapt easily to more open vegetation and to develop a preference for human dwellings over time [33]. In this context, it is important to note that the establishment of agricultural settlements usually involves some degree of deforestation. Crucially, deforestation is strongly linked to an increase in the prevalence of Chagas disease [33]. This connection is supported by the fact that American trypanosomiasis is absent in the indigenous inhabitants of the Amazon region, who used different socio-environmental patterns of land occupation including open communal huts unfavourable for vector colonisation, continuous mobility, and absence of domestic animals which all together hinder vector transmission of Chagas disease [34].

### Modern times

#### 16^th^-19^th^ century

From the 16^th^ century onwards, there are several accounts by travellers and physicians describing patients with disease symptoms reminiscent of American trypanosomiasis. A first suggestive clinical report relating to possible intestinal symptoms of Chagas disease comes from a book published in 1707 by the Portuguese physician Miguel Diaz Pimenta (1661-1715) [35]. Therein he described a condition, which was known as “bicho”, “that causes the humours to be retained, causing the patient to have little desire to eat”. However, a more detailed analysis of the text suggests that the symptoms described refer more likely to haemorrhoids rather than to the clinical picture of a chagasic megacolon [36]. A clearer account on the megavisceral syndrome of Chagas disease comes from another Portuguese physician, Luís Gomes Ferreira (1686-1764), who wrote in 1735 that “the corruption of bicho is nothing else but an enlargement and distension of the rectum” [37,38]. Other records described a condition known then as “mal de engasgo” which probably refers to dysphagia, the difficulty in swallowing [39-41]. For example, the Danish physician Theodoro J. H. Langgaard (1813-1884), who emigrated to Brazil in 1842, gave the following characteristic description of the condition: “…usually the food bolus only passes up to the cardia above the stomach. … Some patients are able to force the descent of the food into the stomach by drinking a small amount of water after each mouthful of ingested food. … As a result of the imperfect nutrition the patients begin to lose weight, become emaciated…” [37,41]. Many more storied references to Chagas disease can be found in an article by Guerra [42]. All these historical accounts indicate that Chagas disease was present in Latin America from the beginning of the 16^th^ century and that it was affecting indigenous people as well as the conquistadors.

There are also many reports of triatomine bugs long before their role as vector for *T. cruzi* was discovered (reviewed in [31] and [37]). Probably the most famous account of a kissing bug comes from Charles Darwin (1809-1882). On the 25^th^ of March 1835 he noted in his diary which he kept during his voyage of The Beagle: “At night I experienced an attack (for it deserves no less a name) of the *Benchuca* (a species of Reduvius) the great black bug of the Pampas. It is most disgusting to feel soft wingless insects, about an inch long, crawling over one’s body. Before sucking they are quite thin, but afterwards become round and bloated with blood, and in this state they are easily crushed. They are also found in the northern part of Chile and in Peru. One which I caught at Iquique was very empty. When placed on the table, and though surrounded by people, if a finger was presented, the bold insect would immediately draw its sucker, make a charge, and if allowed, draw blood. No pain was caused by the wound. It was curious to watch its body during the act of sucking, as it changed in less than ten minutes, from being as flat as a wafer to a globular form. This one feast, for which the benchuca was indebted to one of the officers, kept it fat during four whole months; but, after the first fortnight, the insect was quite ready to have another suck” [43]. Based on this encounter with a kissing bug and his prolonged gastric and nervous symptoms, it was even hypothesised that Darwin was suffering from Chagas disease later in his life. However, Chagas disease is a most unlikely diagnosis for Darwin’s chronic illness as the symptoms abated as he aged, as he did not seem to have any of the typical chagasic symptoms and as he had some of the symptoms already before the Beagle voyage [37]. Despite all these reports, the critical role of triatomine bugs in transmitting Chagas disease remained undiscovered until 1909.

#### 20^th^ century

**Figure 1:**
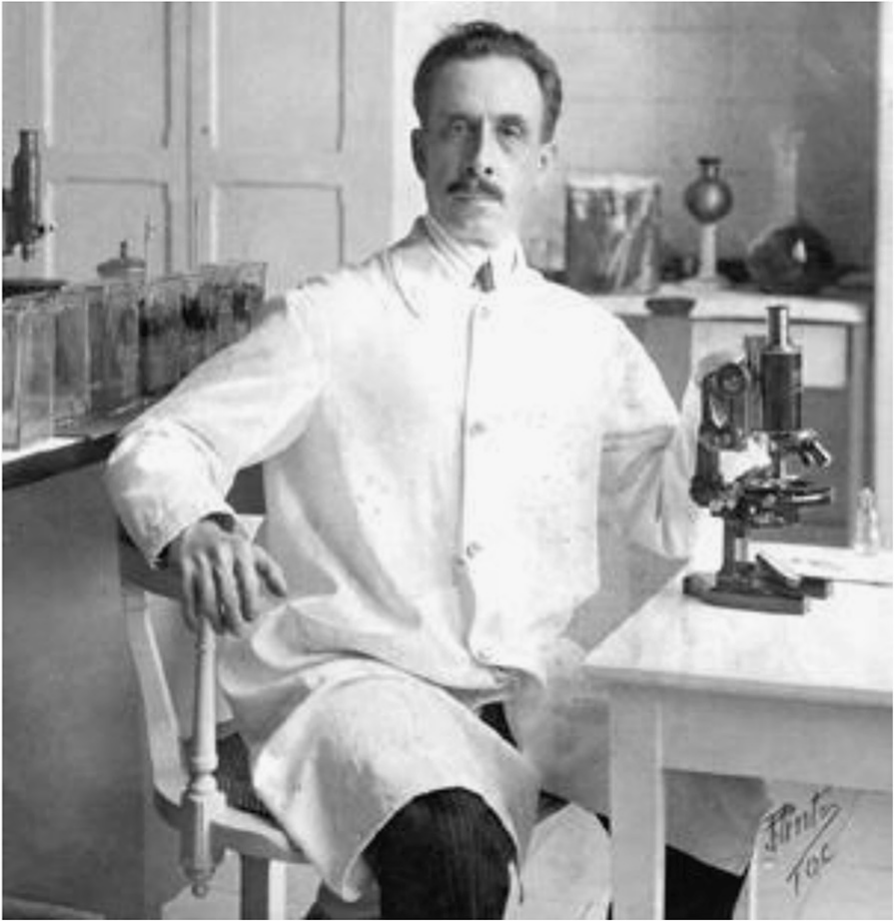
**Carlos Ribeiro Justiniano das Chagas in his laboratory at the Federal Serotherapy Institute in Manguinhos, Rio de Janeiro.** The Brazilian hygienist, scientist and bacteriologist identified the protozoan parasite *T. cruzi* as the causative agent of Chagas disease. Photo taken from Wikimedia Commons.

**Figure 2:**
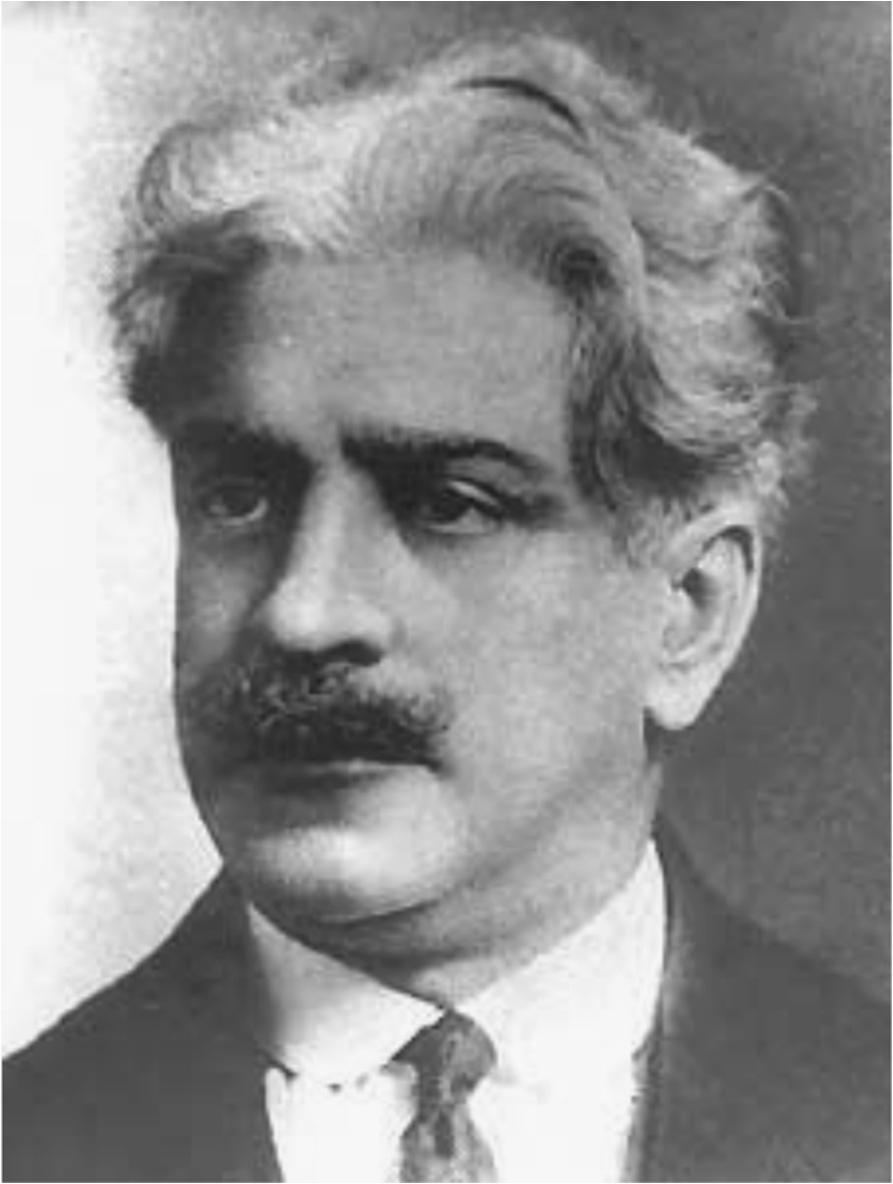
**Oswaldo Gonçalves Cruz.** The Brazilian physician, bacteriologist and epidemiologist was the mentor of Carlos Chagas who discovered American trypanosomiasis. Cruz founded in 1900 the Federal Serotherapy Institute which was renamed in 1905 to Manguinhos Experimental Pathology Institute, then in 1908 to Oswaldo Cruz Institute and finally in 1974 to Oswaldo Cruz Foundation (Fundação Oswaldo Cruz (Fiocruz)). Photo taken from Wikimedia Commons.

In 1908, during an anti-malaria campaign in support of the construction of a railway track in the North of the state of Minas Gerais, the Brazilian hygienist and bacteriologist Carlos Chagas (1879-1934) (Figure 1) was made aware by a railroad engineer of large blood-sucking insects which lived en masse in local dwellings and bit sleeping people preferentially in the face [44]. To see whether these bugs harboured potential pathogens, Chagas dissected them and found numerous trypanosomes in their hindgut which he named *T. cruzi* in honour of his mentor, the Brazilian physician and bacteriologist Oswaldo Cruz (1872-1917) (Figure 2) [45]. Some infected bugs were sent to Cruz in Rio de Janeiro, where they were allowed to bite marmoset monkeys. Within 20-30 days, the monkeys became infected and many trypanosomes were detected in their blood [44]. Soon afterwards Chagas also discovered that the parasite was infective to several other laboratory animals [44]. Chagas was sure that he had found a pathogenic organism of a human infectious disease but did not know what kind of sickness it was. The breakthrough came in 1909 when he was called to examine a two year old girl named Berenice who was feverish with enlarged spleen and liver and swollen lymph nodes [44]. On first examination, no parasites were found but four days later, on the 14^th^ of April 1909, numerous trypanosomes were spotted in her blood with similar morphology to those previously detected in infected marmoset monkeys [44]. Chagas had discovered a new human disease which soon bore his name. He gave a detailed clinical description of the acute phase of the disease and linked the infection with some chronic symptoms of the illness which was remarkable considering that the chronic phase of American trypanosomiasis usually appears decades after the first inoculation with *T. cruzi* (reviewed in [46]). Interestingly, his first patient Berenice never developed determinate chronic Chagas disease and died at the age 73 years on unrelated causes [47]. However, she was infected with *T. cruzi* for her whole life as was confirmed by the isolation of parasites when she was 55 and 71 years old [47]. In 1912, Chagas reported that he had detected *T. cruzi* in an armadillo and thus found the first sylvatic reservoir host [48]. Gradually, more and more sylvatic reservoir animals of Chagas disease were discovered providing evidence for an enzootic cycle of *T. cruzi*.

Without doubt, the discovery of American trypanosomiasis is inseparably linked to Carlos Chagas. However, other researchers have also contributed to the findings. Oswaldo Cruz’s mentoring and the institute he and the Brazilian government founded in 1900 to fight endemic diseases played an important role in the uncovering of Chagas disease [49]. The identification and characterisation of *T. cruzi* was the result of collaboration with the Czech zoologist and parasitologist Stanislaus von Prowazek (1875-1915) who was invited by Cruz to spend six months at the Federal Serotherapy Institute to conduct research (reviewed in [50]). The intracellular life cycle stage of *T. cruzi*, the amastigote form, was described by Chagas’ colleague, the Brazilian pathologist Gaspar de Oliveira Vianna (1885-1914), in both heart and skeletal muscle cells [51]. The mode of transmission of *T. cruzi* was established by the French parasitologist Alexandre Joseph Émile Brumpt (1877-1951) who provided in 1912 clear evidence that the infection resulted not by inoculation but by contamination of the bite wound with parasite containing faeces left behind by the bugs [52].

Chagas’ discovery of a new disease entity brought him worldwide recognition and acknowledgement but also animosity and envy in his own country [53]. This strong opposition not only may have cost him the Nobel Prize, for which he was nominated twice in 1913 and 1921, but also halted the interest in the new disease for almost 20 years [49,53]. The research on Chagas disease resumed in the 1930s when the Argentine physician and epidemiologist Salvador Mazza (1886-1946) described more than a thousand cases in the Argentine Chaco province [54,55]. Mazza was also the first who raised the possibility that Chagas disease could be transmitted by blood transfusion [49]. By the introduction of serodiagnostic tests in the 1940s it was eventually shown that infections with *T. cruzi* were widespread in Latin America [56].

**Figure 3:**
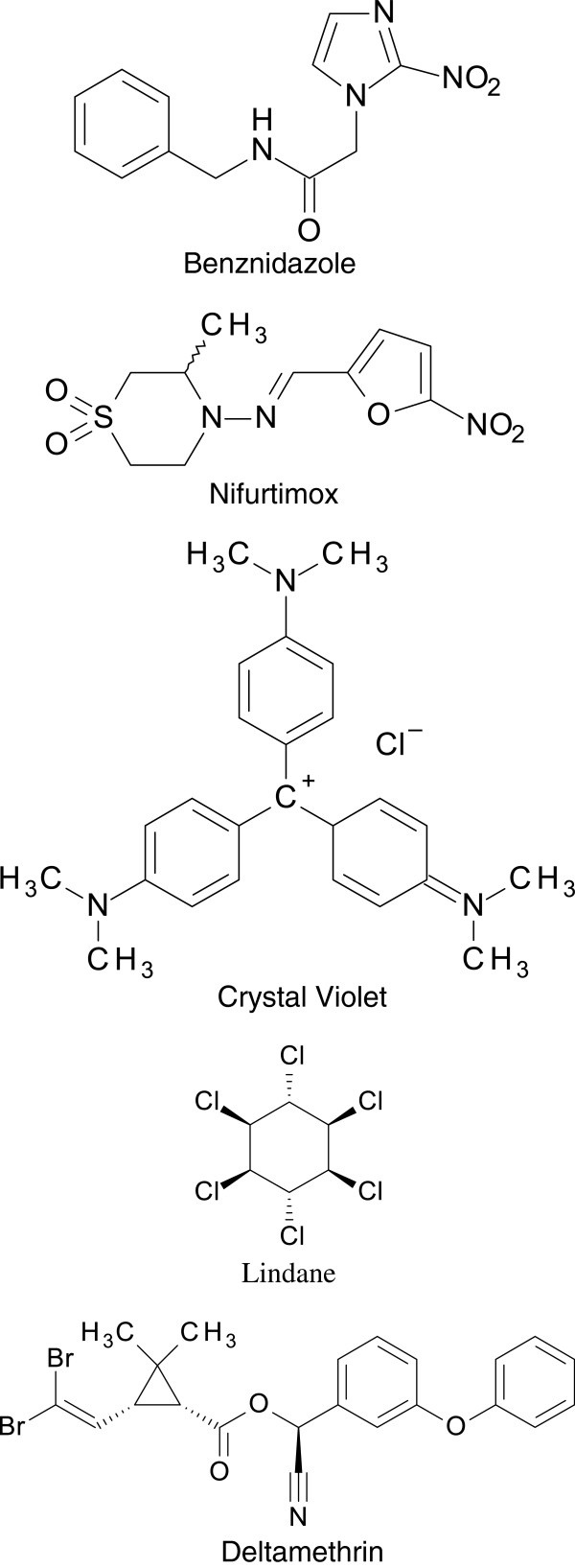
**Chemical structures of compounds used in the treatment and control of Chagas disease.** Benznidazole and nifurtimox are drugs for chemotherapy of acute Chagas disease. Crystal Violet is used in blood banks to eliminate *T. cruzi* from infected human blood. Lindane (an organochlorine) and deltamethrin (a synthetic pyrethroid) are insecticides used in the control of triatomine vectors.

Testing of compounds for treatment of Chagas disease began soon after the discovery of the infection, but without effective results (reviewed in [57]). It took another 50 years before the eventual development of two compounds into drugs for chemotherapy of American trypanosomiasis. In 1966, Hoffmann-La Roche introduced benznidazole (Figure 3) for treatment of Chagas disease [57]. Nifurtimox (Figure 3) was made commercially available as antichagasic medicine by Bayer in 1970 [57]. The production of nifurtimox was suspended in 1997 due to lack of demand, but resumed in 2000 because the compound became part of a new combination therapy for treatment of African sleeping sickness [57,58]. Initially, both drugs were primarily used for treatment of acute cases of Chagas disease because they were considered less effective in the chronic phase [57]. In 1953 it was discovered that the dye crystal violet (aka Gentian Violet; Figure 3) kills *T. cruzi* in blood preservations [59]. Since then the dye has been widely employed in blood banks in endemic areas to eliminate the parasite from blood used for transfusion. Vector control started in the 1940s when the first organochlorine insecticides were developed. Although DTT was quickly found to be ineffective against most triatomine bugs, two other compounds, lindane (Figure 3) and dieldrin were highly effective in killing the vector when sprayed over house walls [60,61]. The introduction of synthetic pyrethroid insecticides (e.g. deltamethrin (Figure 3) and cyfluthrin) in the early 1980s was a major advance in the control of domestic triatomines as they are more cost-effective and leave no unpleasant smell and marks on the treated walls [60,61].

#### Current situation

At present about 7-8 million people are estimated to be infected with *T. cruzi*, mostly in Latin America, and more than 25 million people are at risk of contracting Chagas disease [1,62]. In 2008 alone, more than 10,000 deaths from Chagas disease were reported [62]. Since the 1990s, multinational initiatives have led to significant reductions in the number of acute cases of Chagas disease and in the presence of domestic triatomine vectors in many endemic regions of Latin America [1]. Indoor residual spraying has even lead in the elimination of *R. proxilus* in Central America [63]. Despite these achievements in parasite and vector control, new challenges have arisen. These include recent outbreaks of Chagas disease in the Amazon basin, a region previously thought to be free of the illness, due to oral transmission via contaminated food [64-66], and the ongoing active transmission of Chagas disease in the Bolivian Chaco region in spite of vector control had been in progress there since 2000 [67]. Another problem is the emergence of insecticide resistant triatomine vectors in the Gran Chaco, a region located west of the Paraguay River and east of the Andes [68].

Despite many efforts over the past decades, no drug for the treatment of chronic Chagas disease has been developed so far. However, according to new recommendations published in 2005 and 2007 treatment with nifurtimox and benznidazole is indicated for patients with acute infection as well as for patient < 18 years of age with chronic infection [69,70]. Moreover, treatment should be offered to women in reproductive age as well as to adults < 50 years of age with evidence of chronic infection [69,70]. Treatment is also indicated in immunosuppressed patients with reactivated infection [69,70].

The costs for treatment and prevention of Chagas disease are another current challenge and are a huge burden for the health system of affected countries. For example, in Colombia alone, the costs for medical care of all Chagas patients and for spraying insecticides to vector controls were estimated to be US$ 267 million and US$ 5 million in 2008, respectively [71].

**Figure 4:**
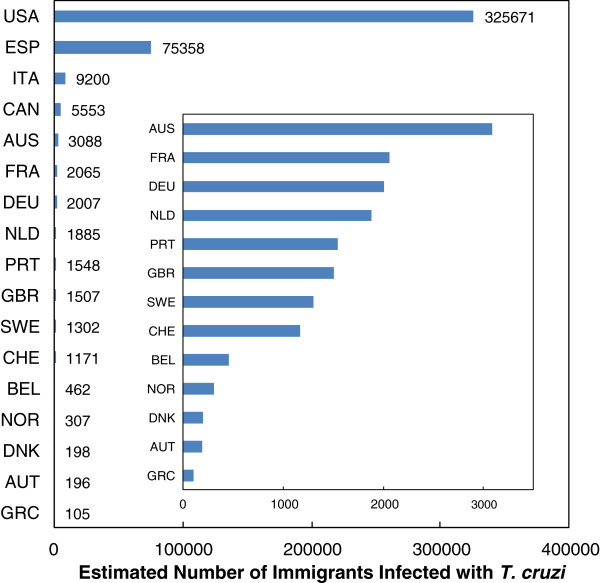
**Estimated number of Chagas cases in non-endemic countries.** Only countries with more than 100 expected cases of *T. cruzi* infection among immigrants were included. The insert shows those countries with less than 5000 expected cases. Data were taken from [70] (United States, Canada and Australia) and [71] (European countries).

Moreover, Chagas disease is increasingly becoming a global health problem. This is due to migration of people infected with *T. cruzi* from endemic countries to North America, Europe and the western Pacific region. The total estimated number of Chagas patients outside Latin America is more than 400,000 with the USA being the most affected country accounting for three-fourths of all cases (Figure 4) [72,73]. In Europe alone, the number of patients that will develop chronic chagasic cardiomyopathy is estimated to be as high as 54,000 [73].

## Conclusion

It is evident from the history of Chagas disease that anthropogenic environmental changes are the primary causes for the transmission of the infection to people. Deforestation seems to be the main factor as it brings people into closer contact with disease-carrying vectors. This is supported by the fact that the transmission of Chagas disease was first induced in ancient times when humans started to clear land for settlements and agriculture. Mining, lumbering and urbanisation are other human activities linked to deforestation which led to an increase in the spread of Chagas disease in more recent times. From the history of Chagas disease one can also learn that triatome vectors have a remarkable ability to quickly adapt to newly created environments and to new hosts. This adaptability of these insects has led to the establishment of domestic transmission cycles between companion animals and humans, which seems to have been crucial for the distribution of Chagas disease throughout Latin America. Through migration Chagas disease has even now become a worldwide health concern.

## Competing interests

The author declares that he has no competing interests.
